# P-1837. Improving Hepatitis B Vaccination Rates in the Ambulatory Care Setting

**DOI:** 10.1093/ofid/ofaf695.2006

**Published:** 2026-01-11

**Authors:** Mya Hnin Lwin, Aparna Rathnam, TuTu Mon, Marlon E Brewer

**Affiliations:** Elmhurst Hospital, Rego Park, NY; Icahn School of Medicine at Mount Sinai, Queens, New York; Elmhurst Hospital, Rego Park, NY; Elmhurst Hospital, Rego Park, NY

## Abstract

**Background:**

Hepatitis B remains a significant public health threat and is one of the leading causes of liver cancer. Vaccination is recommended for all unvaccinated adults aged 19-59. Despite the availability of vaccine, many individuals remain unvaccinated, putting them at risk for severe complications like cirrhosis and hepatocellular carcinoma. The goal of this project was to increase the percentage of patients aged 19-59 in our primary care clinic with negative serology results who receive at least one dose of hepatitis B vaccine from 2% to 30% in one year.

**Figure:**
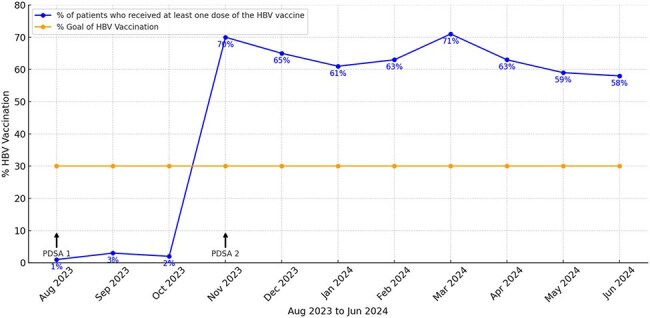
Monthly HBV Vaccination Rates Compared to Goal Improving Hepatitis B Vaccination Rates in the Ambulatory Care Setting

**Methods:**

The full hepatitis serology panel of all unvaccinated individuals within 6 months after screening was reviewed monthly between August 2023 and June 2024 through electronic chart review. Patients with immunity to hepatitis B (indicated by HbsAb or HbcAb) were excluded. The key outcome measure was the percentage of patients aged 19-59 with negative serology results who received at least one dose of hepatitis B vaccine. PDSA cycle 1, in August 2023, providers were emailed about updated hepatitis B vaccination guidelines from the current Center for Disease Control (CDC). For PDSA cycle 2, a multidisciplinary team, including nurses and administrative staffs, was formed in November 2023. The team worked together to identify patients with negative hepatitis B serology and contacted them to schedule vaccination appointments.

**Results:**

PDSA 1 did not have an impact on the percentage of patients receiving the hepatitis B vaccine. The effect of education was limited, primarily because the method of provider education relied on emails. PDSA 2 took a more proactive approach by targeting patient’s awareness about the importance of vaccination and ensuring timely scheduling of vaccination appointments. We reached our goal of 30% after two PDSA cycles and the percentage of patients receiving the Hepatitis B vaccine remained above 55% for the next eight months.

**Conclusion:**

This project highlights the importance of targeted, collaborative efforts to improve hepatitis B vaccination rates in adult primary care setting. By proactively reaching out to patients, we were able to significantly reduce the number of unvaccinated individuals and take a step toward improving public health outcomes.

**Disclosures:**

All Authors: No reported disclosures

